# Comprehensive Phenotype/Genotype Analyses of the Norepinephrine Transporter Gene (SLC6A2) in ADHD: Relation to Maternal Smoking during Pregnancy

**DOI:** 10.1371/journal.pone.0049616

**Published:** 2012-11-20

**Authors:** Geeta A. Thakur, Sarojini M. Sengupta, Natalie Grizenko, Zia Choudhry, Ridha Joober

**Affiliations:** 1 Integrated Program in Neuroscience, McGill University, Montreal, Quebec, Canada; 2 Department of Psychiatry, McGill University, Montreal, Quebec, Canada; 3 Department of Human Genetics, McGill University, Montreal, Quebec, Canada; 4 Douglas Mental Health University Institute, Montreal, Quebec, Canada; Rikagaku Kenkyūsho Brain Science Institute, Japan

## Abstract

**Objective:**

Despite strong pharmacological evidence implicating the norepinephrine transporter in ADHD, genetic studies have yielded largely insignificant results. We tested the association between 30 tag SNPs within the SLC6A2 gene and ADHD, with stratification based on maternal smoking during pregnancy, an environmental factor strongly associated with ADHD.

**Methods:**

Children (6–12 years old) diagnosed with ADHD according to DSM-IV criteria were comprehensively evaluated with regard to several behavioral and cognitive dimensions of ADHD as well as response to a fixed dose of methylphenidate (MPH) using a double-blind placebo controlled crossover trial. Family-based association tests (FBAT), including categorical and quantitative trait analyses, were conducted in 377 nuclear families.

**Results:**

A highly significant association was observed with rs36021 (and linked SNPs) in the group where mothers smoked during pregnancy. Association was noted with categorical DSM-IV ADHD diagnosis (*Z* = 3.74, *P* = 0.0002), behavioral assessments by parents (CBCL, *P* = 0.00008), as well as restless-impulsive subscale scores on Conners’-teachers (*P* = 0.006) and parents (*P* = 0.006). In this subgroup, significant association was also observed with cognitive deficits, more specifically sustained attention, spatial working memory, planning, and response inhibition. The risk allele was associated with significant improvement of behavior as measured by research staff (*Z* = 3.28, *P* = 0.001), parents (*Z* = 2.62, *P* = 0.009), as well as evaluation in the simulated academic environment (*Z* = 3.58, *P* = 0.0003).

**Conclusions:**

By using maternal smoking during pregnancy to index a putatively more homogeneous group of ADHD, highly significant associations were observed between tag SNPs within *SLC6A2* and ADHD diagnosis, behavioral and cognitive measures relevant to ADHD and response to MPH. This comprehensive phenotype/genotype analysis may help to further understand this complex disorder and improve its treatment. Clinical trial registration information – Clinical and Pharmacogenetic Study of Attention Deficit with Hyperactivity Disorder (ADHD); www.clinicaltrials.gov; NCT00483106.

## Introduction

Attention-deficit/hyperactivity disorder (ADHD) is a highly prevalent psychiatric disorder, with rates ranging from 5.9–7.1% in children and adolescents [1]. It is heterogeneous in its clinical expression, with core symptoms of poor sustained attention, impulsivity, and hyperactivity. It is often associated with cognitive deficits, particularly in executive function and sustained attention. ADHD has an important genetic component, with a mean heritability estimate of 76%, [2] and it has been suggested that multiple genes are involved, each having a small effect. [3].

Psychostimulants, mostly methylphenidate (MPH) [4] are the first-line of treatment for ADHD. These medications are known to block the dopamine (DA) and norepinephrine (NE) transporters, resulting in increased synaptic concentration of both neurotransmitters. [5], [6], [7] Short-term trials have concluded that MPH is efficacious in reducing ADHD symptoms in approximately 70% of affected children [4] and adults. [8] NE-specific pharmacological agents (including clonidine, guanfacine, desipramine, and atomoxetine) are effective in treating ADHD, thereby implicating this catecholamine as a major player in the pathophysiology of the disorder. [9] These studies reinforced the early evidence from neurochemical research that NE is involved in ADHD. [10], [11] Neuroimaging [12] and animal studies [13] have provided further evidence for the role of NE in ADHD.

The NE transporter protein is a pivotal player in the regulation of catecholamines, involved in the re-uptake of both NE and DA into presynaptic terminals. Thus, it plays a key role in controlling the intensity and duration of signal transduction. The NE transporter is a member of the sodium- and chloride-dependent neurotransmitter transporter family, a transmembrane glycoprotein. [14] It is encoded by *SLC6A2* which has been mapped to 16 q12.2. [15] The gene includes 14 exons spanning 45 kb, [16] predicting a protein of 617 amino acids. [17] Given the clinical efficacy of agents that block the NE transporter (including psychostimulants, MPH and amphetamine, and the NE-specific agent, atomoxetine), there has been considerable interest in *SLC6A2* as a candidate in genetic and pharmacogenetic studies of ADHD. Importance of the NE transporter has been further emphasized since it is responsible for the reuptake of both NE and DA in the prefrontal cortex (PFC), a brain region critical for attention regulation and where there is a scarcity of the dopamine transporter [18], [19], thus pointing to a potentially greater role of the norepinephrine transporter.

Several family-based [20], [21], [22], [23], [24], [25], [26], [27], [28], [29], [30] and case-control [22], [24], [29], [30], [31], [32], [33] studies have investigated the association between specific polymorphisms within *SLC6A2* and ADHD. While initial studies were conducted with a limited number of single nucleotide polymorphisms (SNPs) [20], [22], [25], [27], recent association studies have used arrays of SNPs covering the entire gene. [21], [23], [26], [29], [30] A number of studies have examined the association between a functional SNP in the promoter region of the gene [−3081(A/T), rs28386840] and ADHD. [24], [28], [31], [32], [33] Furthermore, association between *SLC6A2* and ADHD endophenotypes, including neurocognitive measures [34], [35] or quantitative symptom scores, [36] has also been studied.

Although many studies have been conducted thus far, findings have been limited and difficult to replicate. An earlier study reported an association between rs3785157 and rs998424 and ADHD. [22] Later, an independent group reported a trend for association with both these SNPs, however opposite alleles were conferring risk for the disorder in this study. [30] Although these results were not confirmed in the International Multi-centre ADHD Gene (IMAGE) project, associations were reported with two other SNPs (rs3785143, rs11568324), [23] and these were confirmed in two independent samples. [26], [29] Several related groups have reported an association between ADHD and a functional promoter SNP rs28386840 [−3081(A/T)], using a case-control study design. [31], [32], [33] However, two large family-based studies (one with more than 99% power), conducted by independent groups, failed to replicate this association. [24], [28].

Although several pharmacogenetic studies, including a genome-wide association study, [37] have examined the association between *SLC6A2* SNPs and response to MPH [38], [39], [40] (or OROS-MPH [41], [42]) treatment, only limited association was observed with a few polymorphisms (rs5569, rs28386840, rs17841329, and rs192303) with little replication between studies.

We have conducted a family-based study to test the association between a panel of 30 SNPs within *SLC6A2* and ADHD. In addition to the DSM-IV diagnosis of ADHD, quantitative behavioral and cognitive phenotypes, as well as response of these measures with MPH treatment, were tested for association. The panel of SNPs included those analyzed in the IMAGE project (excluding SNPs having a minor allele frequency ≤0.02), [23] two SNPs selected to extend the 3′ flanking region examined (rs15534, rs7188230), and the functional promoter SNP, rs28386840.

Given that high comorbidity between ADHD and cigarette smoking (35%–45%) is well documented [43], and that children with ADHD are consistently reported to have higher exposure to cigarette smoking during pregnancy compared to the general population (OR = 2.39), [44] analyses were conducted based on stratification by maternal smoking during pregnancy (MSDP). Also, it has been suggested that shared pathways to the two pathologies may exist, at least in some groups of individuals, [45] and more precisely, with respect to monoamine dysregulation. The aim of the current study was to examine the differential association (if any) of genetic polymorphisms within *SLC6A2* after MSDP stratification.

## Methods

### Ethics Statement

The study was approved by the Douglas Mental Health University Institute (DMHUI) Research and Ethics Board. All participating children agreed to take part in the study, and parents provided written consent.

### Subjects

Four hundred and seventy-five children with ADHD between 6 and 12 years of age [mean = 9; SD = 1.8] were included in this study. They were referred by schools, social workers, family doctors and pediatricians, and were recruited from the Disruptive Behavior Disorders Program and the children’s outpatient clinics of the DMHUI, a psychiatric teaching hospital in Montreal, Canada.

Each child was diagnosed with ADHD according to DSM-IV criteria. Further details pertaining to diagnostic procedures have previously been described. [46], [47] Of the total number of affected children, 77.9% were male and 82.5% were of Caucasian ethnicity. 54.1% met DSM-IV criteria for the combined subtype, while 35.6% and 10.3% were diagnosed with the inattentive and hyperactive subtypes, respectively. Among comorbid disorders, 40.6% had oppositional defiant disorder, 22.9% had conduct disorder, 46.2% had anxiety disorder (including specific phobias), and 8.8% had a mood disorder (either/or major depressive episode, dysthymic disorder, manic episode, hypomanic episode).

### Evaluations

The Conners’ Global Index for Parents (Conners’-P) and for Teachers (Conners’-T) [48], [49] were used to evaluate the behavior of the child at home and in the classroom, respectively. The Conners’ Global Index scale has been validated from a genetic point of view, with research showing that genetic factors account for up to 78% of its variance. [50] Parents were also asked to complete the Child Behavior Checklist (CBCL), a comprehensive rating scale (113-item questionnaire) with well-established metric characteristics and representative norms. [51] The raw scores of these scales were transformed into standardized *T* scores with an average of 50; where a score higher than 65 is considered to be clinical. The mean (standard deviation) for the total CBCL, Conners’-P, and Conners’-T scores were: 68.6 (8.9), 73.1 (11.4), and 69.5 (12.7), respectively, in this sample of children. Since it has been shown that a low to moderate correlation exists between parent and teacher reports of ADHD symptoms, and that each may assess a different dimension of the child’s behavior [52], [53], [54], by collecting information from both parents and teachers, a comprehensive assessment of the child’s behavior was obtained.

In addition to clinical dimensions of ADHD, neuropsychological measures, mainly of executive function (EF), were included as quantitative traits in the genetic association analyses. EF encapsulates the range of cognitive abilities that are important for self-regulation and goal-directed behaviors, including response inhibition, sustained attention, working memory, set-shifting, planning, and organization. Deficits in EF have been implicated in the underlying pathophysiology of ADHD. [55] The following tests were included in the neuropsychological battery: Wisconsin Card Sorting Test (WCST; measure of cognitive flexibility and set-shifting), [56] Tower of London test (TOL; planning, organization, and problem-solving capacity), [57] Self-Ordered Pointing Task (SOPT; visual working memory, planning and response inhibition), [58] Conners’ Continuous Performance Test (CPT; attention, response inhibition, and impulse control)_ENREF_57 [59] and Finger Windows (FW; visual-spatial working memory). [60] The WCST, TOL, SOPT, and CPT were performed as described elsewhere. [61], [62] FW is a subtest of the Wide Range Assessment of Memory and Learning (WRAML). In this test, the child is required to repeat a sequential placement of a pencil into a series of holes on a plastic card, as conducted by the examiner. When children were medicated prior to their inclusion in the study, clinical and neuropsychological assessments were carried out at the end of a one-week washout period to limit variability due to medication effects. [63] In addition to these EF measures, IQ (full scale, verbal, and performance) was evaluated using the Wechsler Intelligence Scale (WISC-III/IV). [64].

Response to treatment with methylphenidate (MPH) was assessed in a double-blind, placebo-controlled, within-subject (crossover) randomized control trial conducted over a two-week period, as described (trial registration number: NCT00483106). [46] Following a one-week wash-out period, subjects received either one week of treatment with placebo (PBO) or one week of treatment with 0.5 mg/kg of MPH in a divided b.i.d. dose (0.25 mg/kg, morning and noon), and were then crossed over. At the end of each treatment week, parents and teacher were asked to evaluate the child’s behavior using the Conners’-P and Conners’-T, respectively. Assessments were performed before and after the administration of PBO and MPH. In addition, the clinical staff completed the Clinical Global Impression (CGI)-overall improvement scale based on their half day of behavioral observation while the child was completing various tasks in the clinic. In this study, MPH was used as a pharmacological probe to dynamically study the genetics of ADHD, rather than a classical trial of response to medication.

The Restricted Academic Situation Scale (RASS) was used to assess task-oriented behavior. During a simulated independent academic situation within a clinic setting [65], the child is assigned a set of math problems and the RASS (coding system) is used to record the child’s behavior as well as his or her ability for sustained attention to routine, repetitive academic work in the presence of potential distractions, with no adult supervision. [66] The task has previously been described in detail. [67] Over a 15 minute period, behavioral events are recorded at 30 second intervals, according to five categories: *off-task* (looking away from the task sheet), *playing with objects* (touching any object not directly used in the task), *out of seat* (lifting buttocks off chair or moving chair), *vocalizing* (any vocal noise, whether task related or not), and *fidgeting* (repetitive, purposeless movements). The RASS score is the total number of recorded behavioral events, and the difference score is obtained by subtracting the score after MPH administration from the score obtained after PBO. We have previously reported results from principal component analysis of the RASS [68] showing that off-task, out-of-seat, and playing with objects consist of one factor, while vocalizing and fidgeting appear to be independent factors.

### Genotyping

Families were invited to participate in the genetic component of the study, where DNA was extracted, for each parent and child, from a blood sample, buccal swab, or saliva sample, if the subject was only amenable to the latter. Of the 377 nuclear families with one or more children diagnosed with ADHD, 184 were complete trios with information from both parents, 11 were trios with two affected children, 67 were trios with information from one parent and one or more unaffected sibling, 103 were duos including the proband and one parent, while 12 were families with two affected siblings and one parent.

Tag SNPs within *SLC6A2*, previously examined in the IMAGE project, were genotyped [23]. Those with a very low minor allele frequency (MAF ≤0.02) were excluded, with one exception: rs11568324 (MAF = 0.01), since this SNP was shown to be associated with ADHD in the original IMAGE study [23] and in a subsequent replication study. [26] Another SNP (rs28386840) which encodes a functional polymorphism in the upstream promoter region of *SLC6A2*, was also included in the panel, since it has been associated with ADHD [32]. In order to extend the flanking region examined in *SLC6A2*, two SNPs (rs15534, present in exon 14; rs7188230, present in the 3′ intergenic region) not genotyped in the IMAGE study, were also included in this study.

Sequenom iPlex Gold Technology [69] was used to genotype the panel of SNPs, where each plate included duplicates of two reference samples to estimate genotyping error. Genotypes for these samples were read with 100% accuracy on each of the plates. Five SNPs in the original panel in the IMAGE study (rs7201099, rs3760019, rs1362620, rs1861647, rs1566652) could not be genotyped on the Sequenom platform. Since these SNPs were in strong linkage disequilibrium (LD) with other SNPs in the panel, and were not shown to be specifically associated in any previous studies, they were excluded from subsequent analyses. The genotype distribution at each of the markers analyzed in this study did not depart from Hardy-Weinberg equilibrium. [47] By using genotype information from the current study [70] and the default definition in Haploview [71], an LD plot was generated in Haploview v4.0. In this method, 95% confidence bounds on D’ are generated for each pairwise comparison. A SNP block is formed if 95% of the informative comparisons are in strong LD with each other. As indicated by the color coded cells seen in Tables 1–6 and 8–10, three major haplotype blocks exist in *SLC6A2*.

**Table 1:** Association between *SLC6A2* SNPs and ADHD behavioural dimensions in the total sample.

			BLOCK 1														BLOCK 2										BLOCK 3			
TAG SNPs/HAPLOTYPES	rs28386840	rs4783899	rs1362621	rs2397771	rs168924	rs2242446	rs3785143	rs192303	rs41154	rs187715	rs36024	rs187714	rs36023	rs36021	rs3785152	rs1814269	rs36017	rs10521329	rs3785155	rs5564	rs11568324	rs2279805	rs8047672	rs5569	rs998424	rs36009	rs1800887	rs2242447	rs15534	rs7188230
ALLELES	A	T	A	G	T	T	C	G	G	A	C	C	C	T	C	A	G	C	G	T	C	C	G	C	C	G	T	T	C	A
BEHAVIOURAL DIMENSIONS																														
ADHD							+1		+1					+1																
Total # of DISC ADHD items		+1		+1			+1		+1					+1																
# of DISC inattentive items		+1		+2			+1		+1					+1										+1	+1					
# of DISC hyperactivity items														+1																
# of DISC impulsivity items																														
# of DISC ODD items							+1							+1																
# of DISC CD items																														
CBCL Total score							+2							+1		+1						+1								
CBCL Internalizing behaviour							+2							+1	+1	+1						+1								
CBCL Externalizing behaviour							+2							+1																
CBCL Withdrawn				+1			+2		+1					+1													+1	+1		+1
CBCL Somatic complaints							+2							+1	+1															
CBCL Anxious/depressed		+1		+1			+2		+2			+1		+1		+1	+1					+1					+1		+1	+1
CBCL Social problems		+1		+2			+1		+2			+1		+2													+1			
CBCL Thought problems							+2							+1			+2					+2		+1	+1		+1	+1		+1
CBCL Attention problems				+1			+2							+1																
CBCL Delinquent behaviour																														
CBCL Aggressive behaviour							+2							+1																+1
Conners’ P RI Baseline																								+1	+1					
Conners’ T RI Baseline																														
Conners’ P EL Baseline																														
Conners’ T EL Baseline																														
Conners’ P Baseline																								+1	+1					
Conners’ T Baseline																														

ADHD = Attention-Deficit/Hyperactivity Disorder, DISC = Diagnostic Interview Schedule for Children, ODD = Oppositional Defiant Disorder, CD = Conduct Disorder, CBCL = Child Behaviour Checklist, Conners’ P = Conners’ Parents, Conners’ T = Conners’ Teachers, RI = Restless-Impulsive, EL = Emotional Lability.

Three major haplotype blocks in *SLC6A2* are depicted above: Block 1 (rs1362621, rs2397771, rs168924, rs2242446, rs3785143, rs192303, rs41154); Block 2 (rs36017, rs10521329, rs3785155, rs5564, rs11568324, rs2279805, rs8047672, rs5569, rs998424, rs36009); and Block 3 (rs1800887, rs2242447, rs15534).

Significance (p) value ranges are depicted as follows: For OVER-TRANSMISSION of alleles = **+4** (≤0.00001), **+3** (0.0001–0.0009), **+2** (0.001–0.009), and **+1** (0.01–0.049).

For UNDER-TRANSMISSION of alleles = −**4** (≤0.00001), −**3** (0.0001–0.0009), −**2** (0.001–0.009) and −**1** (0.01–0.049).

**Table 2:** Association between *SLC6A2* SNPs and ADHD cognitive dimensions in the total sample.

			BLOCK 1														BLOCK 2										BLOCK 3			
TAG SNPs/HAPLOTYPES	rs28386840	rs4783899	rs1362621	rs2397771	rs168924	rs2242446	rs3785143	rs192303	rs41154	rs187715	rs36024	rs187714	rs36023	rs36021	rs3785152	rs1814269	rs36017	rs10521329	rs3785155	rs5564	rs11568324	rs2279805	rs8047672	rs5569	rs998424	rs36009	rs1800887	rs2242447	rs15534	rs7188230
ALLELES	A	T	A	G	T	T	C	G	G	A	C	C	C	T	C	A	G	C	G	T	C	C	G	C	C	G	T	T	C	A
COGNITIVE DIMENSIONS																														
WISC IQ															−1	−2											−1	−1		
WISC Verbal IQ														−1	−1	−1														
WISC Performance IQ																														
SOPT Total score							+1							+1													+1	+1		
FW Score							+1																							
CPT Overall index														+2													+2	+2		+1
CPT Omission errors														+1																
CPT Commission errors																+1												+1		
CPT Hit Reaction Time																														
CPT Hit Reaction Time SE														+1																
CPT Variability of SE														+1																
CPT Detectability																												+1		
CPT Response Style																											+1			
CPT Perseveration														+1						+1								+1		
CPT Hit reaction time block change			+1				+1																							
CPT Hit SE block change																														
CPT Hit RT ISI change														+1													+1	+1		+1
CPT Hit SE ISI change																														
WCST Total errors standard score								−1																		−1		−1		
WCST Perseverative responses																										−1				
WCST Perseverative errors																														
WCST Non-perseverative errors								−1						−1														−1		−1
TOL Standard score																														

WISC = Wechsler Intelligence Scale, SOPT = Self-Ordered Pointing Task, FW = Finger Windows, CPT = Continuous Performance Test, SE = standard error, RT = reaction time, ISI = inter-stimulus interval, WCST = Wisconsin Card Sorting Test, TOL = Tower of London.

Standard scores were used for all WCST and TOL measures, and T-scores were used for CPT measures (excl. overall index).

Three major haplotype blocks in *SLC6A2* are depicted above: Block 1 (rs1362621, rs2397771, rs168924, rs2242446, rs3785143, rs192303, rs41154); Block 2 (rs36017, rs10521329, rs3785155, rs5564, rs11568324, rs2279805, rs8047672, rs5569, rs998424, rs36009); and Block 3 (rs1800887, rs2242447, rs15534).

Significance (p) value ranges are depicted as follows: For OVER-TRANSMISSION of alleles = **+4** (≤0.00001), **+3** (0.0001–0.0009), **+2** (0.001–0.009), and **+1** (0.01–0.049).

For UNDER-TRANSMISSION of alleles = −**4** (≤0.00001), −**3** (0.0001–0.0009), −**2** (0.001–0.009) and −**1** (0.01–0.049).

**Table 3:** Association between *SLC6A2* SNPs and treatment response in the total sample.

			BLOCK 1														BLOCK 2										BLOCK 3			
TAG SNPs/HAPLOTYPES	rs28386840	rs4783899	rs1362621	rs2397771	rs168924	rs2242446	rs3785143	rs192303	rs41154	rs187715	rs36024	rs187714	rs36023	rs36021	rs3785152	rs1814269	rs36017	rs10521329	rs3785155	rs5564	rs11568324	rs2279805	rs8047672	rs5569	rs998424	rs36009	rs1800887	rs2242447	rs15534	rs7188230
ALLELES	A	T	A	G	T	T	C	G	G	A	C	C	C	T	C	A	G	C	G	T	C	C	G	C	C	G	T	T	C	A
TREATMENT RESPONSE																														
CGI - improvement (PBO-active)							+1							+1																
Conners’ P RI (PBO-active)																											+1			
Conners’ T RI (PBO-active)																									+1	+1				
Conners’ P EL (PBO-active)		+1		+1																										
Conners’ T EL (PBO-active)																			−1	+2						+1				
Conners’ P (PBO-active)																											+1			
Conners’ T (PBO-active)																				+1						+1				
RASS total difference score (PBO time2-Active time2)														+1	+2	+1											+1			+1
RASS fidgeting difference score								+1	+1				+1				+1		+1			+1					+1		+2	+1
RASS vocalization difference score					−1					−1			−1		+1															
RASS task disengagement difference score														+1	+2	+1														

CGI = Clinical Global Impression, PBO = placebo, Conners’ P = Conners’ Parents, Conners’ T = Conners’ Teachers, RI = Restless-Impulsive, EL = Emotional Lability, RASS = Restricted Academic Situation Scale.

Three major haplotype blocks in *SLC6A2* are depicted above: Block 1 (rs1362621, rs2397771, rs168924, rs2242446, rs3785143, rs192303, rs41154); Block 2 (rs36017, rs10521329, rs3785155, rs5564, rs11568324, rs2279805, rs8047672, rs5569, rs998424, rs36009); and Block 3 (rs1800887, rs2242447, rs15534).

Significance (p) value ranges are depicted as follows: For OVER-TRANSMISSION of alleles = **+4** (≤0.00001), **+3** (0.0001–0.0009), **+2** (0.001–0.009), and **+1** (0.01–0.049).

For UNDER-TRANSMISSION of alleles = −**4** (≤0.00001), −**3** (0.0001–0.0009), −**2** (0.001–0.009) and −**1** (0.01–0.049).

**Table 4:** Association between *SLC6A2* SNPs and ADHD behavioural dimensions in the group where mothers smoked during pregnancy (MSDP).

			BLOCK 1														BLOCK 2									BLOCK 3			
TAG SNPs/HAPLOTYPES	rs28386840	rs4783899	rs1362621	rs2397771	rs168924	rs2242446	rs3785143	rs192303	rs41154	rs187715	rs36024	rs187714	rs36023	rs36021	rs3785152	rs1814269	rs36017	rs10521329	rs3785155	rs5564	rs2279805	rs8047672	rs5569	rs998424	rs36009	rs1800887	rs2242447	rs15534	rs7188230
ALLELES	A	T	A	G	T	T	C	G	G	A	C	C	C	T	T	A	G	C	G	T	C	G	A	T	G	T	T	C	A
**BEHAVIOURAL DIMENSIONS**																													
ADHD		**+1**		**+1**				**+1**	**+2**			**+2**		**+3**															
Total # of DISC ADHD items		**+1**		**+1**				**+1**	**+2**			**+2**		**+3**															
# of DISC inattentive items		**+2**		**+1**				**+1**	**+3**		**+1**	**+2**		**+4**															
# of DISC hyperactivity items								**+1**	**+2**			**+1**		**+3**															
# of DISC impulsivity items								**+1**	**+1**			**+1**		**+2**															
# of DISC ODD items									**+1**			**+1**		**+2**															
# of DISC CD items														**+1**															
CBCL Total score							**+1**	**+1**	**+2**			**+1**		**+4**												**+1**			**+1**
CBCL Internalizing behaviour								**+1**	**+1**			**+1**		**+3**															**+1**
CBCL Externalizing behaviour							**+1**	**+1**	**+2**			**+1**		**+3**															
CBCL Withdrawn									**+2**			**+1**		**+3**												**+1**			**+1**
CBCL Somatic complaints								**+1**	**+1**			**+1**		**+3**															
CBCL Anxious/depressed		**+2**		**+1**				**+1**	**+3**			**+2**		**+3**												**+1**		**+1**	**+1**
CBCL Social problems		**+1**		**+1**				**+2**	**+3**			**+2**		**+3**					**+1**							**+1**			
CBCL Thought problems								**+1**	**+1**					**+3**												**+1**	**+1**	**+1**	**+2**
CBCL Attention problems		**+1**		**+1**			**+1**	**+1**	**+2**			**+2**		**+3**					**+1**							**+1**	**+1**	**+1**	**+1**
CBCL Delinquent behaviour														**+1**															
CBCL Aggressive behaviour							**+1**	**+1**	**+1**			**+1**		**+3**												**+1**			**+1**
Conners’ P RI Baseline									**+1**					**+2**															
Conners’ T RI Baseline							**+1**							**+2**															
Conners’ P EL Baseline		**+1**						**+1**	**+1**			**+1**	**+1**	**+1**															
Conners’ T EL Baseline																													
Conners’ P Baseline		**+1**						**+1**	**+1**			**+1**		**+2**															
Conners’ T Baseline														**+1**															

ADHD = Attention-Deficit/Hyperactivity Disorder, DISC = Diagnostic Interview Schedule for Children, ODD = Oppositional Defiant Disorder, CD = Conduct Disorder, CBCL = Child Behaviour Checklist, Conners’ P = Conners’ Parents, Conners’ T = Conners’ Teachers, RI = Restless-Impulsive, EL = Emotional Lability.

Three major haplotype blocks in *SLC6A2* are depicted above: Block 1 (rs1362621, rs2397771, rs168924, rs2242446, rs3785143, rs192303, rs41154); Block 2 (rs36017, rs10521329, rs3785155, rs5564, rs11568324, rs2279805, rs8047672, rs5569, rs998424, rs36009); and Block 3 (rs1800887, rs2242447, rs15534).

Significance (p) value ranges are depicted as follows: For OVER-TRANSMISSION of alleles = **+4** (≤0.00001), **+3** (0.0001–0.0009), **+2** (0.001–0.009), and **+1** (0.01–0.049).

For UNDER-TRANSMISSION of alleles = −**4** (≤0.00001), −**3** (0.0001–0.0009), −**2** (0.001–0.009) and −**1** (0.01–0.049).

**Table 5:** Association between *SLC6A2* SNPs and ADHD cognitive dimensions in the MSDP group.

			BLOCK 1														BLOCK 2									BLOCK 3			
TAG SNPs/HAPLOTYPES	rs28386840	rs4783899	rs1362621	rs2397771	rs168924	rs2242446	rs3785143	rs192303	rs41154	rs187715	rs36024	rs187714	rs36023	rs36021	rs3785152	rs1814269	rs36017	rs10521329	rs3785155	rs5564	rs2279805	rs8047672	rs5569	rs998424	rs36009	rs1800887	rs2242447	rs15534	rs7188230
ALLELES	A	T	A	G	T	T	C	G	G	A	C	C	C	T	T	A	G	C	G	T	C	G	A	T	G	T	T	C	A
**COGNITIVE DIMENSIONS**																													
WISC IQ																−**2**		−**1**				−**1**				−**1**	−**2**	−**1**	−**1**
WISC Verbal IQ														−**1**		−**1**						−**1**				−**1**	−**1**		
WISC Performance IQ																													
SOPT Total score							**+1**	**+1**	**+1**			**+1**		**+3**					**+1**							**+2**	**+1**	**+1**	**+1**
FW Score									**+1**			**+1**		**+2**															
CPT Overall index									**+1**			**+1**		**+3**												**+1**	**+1**		**+1**
CPT Omission errors																													
CPT Commission errors		**+1**																								**+1**			
CPT Hit Reaction Time									**+1**					**+1**															
CPT Hit Reaction Time SE									**+1**			**+1**		**+3**															
CPT Variability of SE														**+2**															
CPT Detectability																										**+1**			
CPT Response Style																										**+1**		**+1**	
CPT Perseveration									**+1**			**+1**		**+1**												**+1**	**+2**		**+1**
CPT Hit reaction time block change																										**+1**	**+1**		**+1**
CPT Hit SE block change																													
CPT Hit RT ISI change							**+1**		**+1**			**+1**		**+3**												**+1**			
CPT Hit SE ISI change							**+1**							**+2**															
WCST Total errors standard score								−**1**						−**2**															−**1**
WCST Perseverative responses																													
WCST Perseverative errors																													
WCST Non-perseverative errors								−**1**						−**3**													−**1**		−**1**
TOL Standard score																							**+1**						

WISC = Wechsler Intelligence Scale, SOPT = Self-Ordered Pointing Task, FW = Finger Windows, CPT = Continuous Performance Test, SE = standard error, RT = reaction time, ISI = inter-stimulus interval, WCST = Wisconsin Card Sorting Test, TOL = Tower of London.

Standard scores were used for all WCST and TOL measures, and T-scores were used for CPT measures (excl. overall index).

Three major haplotype blocks in *SLC6A2* are depicted above: Block 1 (rs1362621, rs2397771, rs168924, rs2242446, rs3785143, rs192303, rs41154); Block 2 (rs36017, rs10521329, rs3785155, rs5564, rs11568324, rs2279805, rs8047672, rs5569, rs998424, rs36009); and Block 3 (rs1800887, rs2242447, rs15534).

Significance (p) value ranges are depicted as follows: For OVER-TRANSMISSION of alleles = **+4** (≤0.00001), **+3** (0.0001–0.0009), **+2** (0.001–0.009), and **+1** (0.01–0.049).

For UNDER-TRANSMISSION of alleles = −**4** (≤0.00001), −**3** (0.0001–0.0009), −**2** (0.001–0.009) and −**1** (0.01–0.049).

**Table 6:** Association between *SLC6A2* SNPs and treatment response in the MSDP group.

			BLOCK 1														BLOCK 2									BLOCK 3			
TAG SNPs/HAPLOTYPES	rs28386840	rs4783899	rs1362621	rs2397771	rs168924	rs2242446	rs3785143	rs192303	rs41154	rs187715	rs36024	rs187714	rs36023	rs36021	rs3785152	rs1814269	rs36017	rs10521329	rs3785155	rs5564	rs2279805	rs8047672	rs5569	rs998424	rs36009	rs1800887	rs2242447	rs15534	rs7188230
ALLELES	A	T	A	G	T	T	C	G	G	A	C	C	C	T	T	A	G	C	G	T	C	G	A	T	G	T	T	C	A
**TREATMENT RESPONSE**																													
CGI - improvement (PBO-active)									**+1**					**+2**												**+1**			**+1**
Conners’ P RI (PBO-active)		**+1**		**+1**										**+1**															
Conners’ T RI (PBO-active)	**+1**											**+1**		**+1**															
Conners’ P EL (PBO-active)									**+1**					**+1**				**+1**	**+1**			**+1**				**+1**			
Conners’ T EL (PBO-active)																													
Conners’ P (PBO-active)		**+1**		**+1**					**+1**					**+2**				**+1**											
Conners’ T (PBO-active)														**+1**															
RASS total difference score (PBO time2-Active time2)									**+1**			**+1**		**+3**															
RASS fidgeting difference score																												**+1**	
RASS vocalization difference score							**+1**			−**1**				**+1**															
RASS task disengagement difference score		**+1**							**+1**					**+3**												**+1**			

CGI = Clinical Global Impression, PBO = placebo, Conners’ P = Conners’ Parents, Conners’ T = Conners’ Teachers, RI = Restless-Impulsive, EL = Emotional Lability, RASS = Restricted Academic Situation Scale.

Three major haplotype blocks in *SLC6A2* are depicted above: Block 1 (rs1362621, rs2397771, rs168924, rs2242446, rs3785143, rs192303, rs41154); Block 2 (rs36017, rs10521329, rs3785155, rs5564, rs11568324, rs2279805, rs8047672, rs5569, rs998424, rs36009); and Block 3 (rs1800887, rs2242447, rs15534).

Significance (p) value ranges are depicted as follows: For OVER-TRANSMISSION of alleles = **+4** (≤0.00001), **+3** (0.0001–0.0009), **+2** (0.001–0.009), and **+1** (0.01–0.049).

For UNDER-TRANSMISSION of alleles = −**4** (≤0.00001), −**3** (0.0001–0.0009), −**2** (0.001–0.009) and −**1** (0.01–0.049).

**Table 7:** Linkage disequilibrium between *SLC6A2* markers.

LD with rs36021	D’	r-square	LD with rs3785152	D’	r-square
rs28386840	0,234	0,031	rs28386840	0,221	0,003
*rs4783899*	0,596	0,266	rs4783899	0,181	0,005
rs1362621	0,209	0,023	rs1362621	0,043	0
*rs2397771*	0,35	0,101	rs2397771	0,154	0,002
rs168924	0,659	0,096	rs168924	0,438	0,004
rs2242446	0,219	0,025	rs2242446	0,046	0
rs3785143	1	0,136	rs3785143	0,889	0,01
*rs192303*	0,78	0,333	rs192303	0,542	0,016
*rs41154*	0,919	0,431	rs41154	0,241	0,011
rs187715	0,958	0,05	rs187715	0,183	0
rs36024	0,333	0,109	rs36024	0,08	0,001
*rs187714*	0,917	0,445	rs187714	0,338	0,022
rs36023	0,202	0,03	rs36023	0,274	0,006
rs36021			rs36021	0,418	0,018
rs3785152	0,418	0,018	rs3785152		
rs1814269	0,091	0,004	rs1814269	0,483	0,021
rs36017	0,124	0,01	rs36017	0,152	0,003
rs10521329	0,296	0,023	rs10521329	0,033	0
rs3785155	0,386	0,026	rs3785155	0,511	0,005
rs5564	0,572	0,014	rs5564	0,535	0,123
rs11568324	1	0,009	rs11568324	1	0,002
rs2279805	0,143	0,014	rs2279805	0,009	0
rs8047672	0,29	0,022	rs8047672	0,071	0
rs5569	0,002	0	rs5569	0,133	0,001
rs998424	0,002	0	rs998424	0,133	0,001
rs36009	0,444	0,011	rs36009	0,272	0,04
rs1800887	0,239	0,021	rs1800887	0,035	0,001
rs2242447	0,083	0,005	rs2242447	0,392	0,039
rs15534	0,319	0,025	rs15534	0,029	0,001
rs7188230	0,235	0,02	rs7188230	0,022	0

Five SNPs towards the 5′end of *SLC6A2* (rs41154, rs187714, rs4783899, rs2397771, rs192303) are in strong linkage disequilibrium (LD) with rs36021. LD = linkage disequilibrium

**Table 8:** Association between *SLC6A2* SNPs and ADHD behavioural dimensions in the sample where mothers did not smoke during pregnancy.

			BLOCK 1														BLOCK 2									BLOCK 3			
TAG SNPs/HAPLOTYPES	rs28386840	rs4783899	rs1362621	rs2397771	rs168924	rs2242446	rs3785143	rs192303	rs41154	rs187715	rs36024	rs187714	rs36023	rs36021	rs3785152	rs1814269	rs36017	rs10521329	rs3785155	rs5564	rs2279805	rs8047672	rs5569	rs998424	rs36009	rs1800887	rs2242447	rs15534	rs7188230
ALLELES	A	T	A	G	C	T	C	C	G	A	C	C	C	T	C	A	G	A	A	T	C	A	G	C	G	T	T	T	A
**BEHAVIOURAL DIMENSIONS**																													
ADHD																							**+1**	**+1**					
Total # of DISC ADHD items															**+1**								**+1**	**+1**					
# of DISC inattentive items															**+1**								**+2**	**+1**					
# of DISC hyperactivity items															**+1**														
# of DISC impulsivity items																									**+1**				
# of DISC ODD items							**+1**																						
# of DISC CD items																													
CBCL Total score							**+1**								**+1**	**+1**													
CBCL Internalizing behaviour							**+1**								**+1**	**+1**													
CBCL Externalizing behaviour							**+1**								**+1**														
CBCL Withdrawn							**+1**									**+1**													
CBCL Somatic complaints							**+1**								**+2**														
CBCL Anxious/depressed							**+1**									**+2**	**+1**												
CBCL Social problems															**+1**														
CBCL Thought problems							**+1**								**+2**		**+1**				**+1**		**+1**	**+1**					
CBCL Attention problems							**+1**								**+1**								**+1**	**+1**					
CBCL Delinquent behaviour																													
CBCL Aggressive behaviour															**+1**														
Conners’ P RI Baseline															**+1**								**+2**	**+2**					
Conners’ T RI Baseline																							**+1**	**+1**					
Conners’ P EL Baseline															**+1**														
Conners’ T EL Baseline																													
Conners’ P Baseline															**+2**								**+1**	**+1**					
Conners’ T Baseline																													

ADHD = Attention-Deficit/Hyperactivity Disorder, DISC = Diagnostic Interview Schedule for Children, ODD = Oppositional Defiant Disorder, CD = Conduct Disorder, CBCL = Child Behaviour Checklist, Conners’ P = Conners’ Parents, Conners’ T = Conners’ Teachers, RI = Restless-Impulsive, EL = Emotional Lability.

Three major haplotype blocks in *SLC6A2* are depicted above: Block 1 (rs1362621, rs2397771, rs168924, rs2242446, rs3785143, rs192303, rs41154); Block 2 (rs36017, rs10521329, rs3785155, rs5564, rs11568324, rs2279805, rs8047672, rs5569, rs998424, rs36009); and Block 3 (rs1800887, rs2242447, rs15534).

Significance (p) value ranges are depicted as follows: For OVER-TRANSMISSION of alleles = **+4** (≤0.00001), **+3** (0.0001–0.0009), **+2** (0.001–0.009), and **+1** (0.01–0.049).

For UNDER-TRANSMISSION of alleles = −**4** (≤0.00001), −**3** (0.0001–0.0009), −**2** (0.001–0.009) and −**1** (0.01–0.049).

**Table 9:** Association between *SLC6A2* SNPs and ADHD cognitive dimensions in the sample where mothers did not smoke during pregnancy.

			BLOCK 1														BLOCK 2									BLOCK 3			
TAG SNPs/HAPLOTYPES	rs28386840	rs4783899	rs1362621	rs2397771	rs168924	rs2242446	rs3785143	rs192303	rs41154	rs187715	rs36024	rs187714	rs36023	rs36021	rs3785152	rs1814269	rs36017	rs10521329	rs3785155	rs5564	rs2279805	rs8047672	rs5569	rs998424	rs36009	rs1800887	rs2242447	rs15534	rs7188230
ALLELES	A	T	A	G	C	T	C	C	G	A	C	C	C	T	C	A	G	A	A	T	C	A	G	C	G	T	T	T	A
**COGNITIVE DIMENSIONS**																													
WISC IQ															−**1**														
WISC Verbal IQ															−**1**														
WISC Performance IQ																													
SOPT Total score																							**+1**	**+1**					
FW Score																													
CPT Overall index																				**+1**					**+2**				
CPT Omission errors																													
CPT Commission errors																							**+1**	**+1**					
CPT Hit Reaction Time		−**1**								−**1**									−**1**	**+1**									
CPT Hit Reaction Time SE																													
CPT Variability of SE																													
CPT Detectability															**+2**	**+1**			**+1**				**+1**	**+1**					
CPT Response Style																**+1**													
CPT Perseveration																				**+1**					**+1**				
CPT Hit reaction time block change																						**+1**							
CPT Hit SE block change																													
CPT Hit RT ISI change																									**+1**				
CPT Hit SE ISI change																									**+1**				
WCST Total errors standard score																									−**2**		−**1**		
WCST Perseverative responses																									−**2**				
WCST Perseverative errors																													
WCST Non-perseverative errors																													
TOL Standard score																													

WISC = Wechsler Intelligence Scale, SOPT = Self-Ordered Pointing Task, FW = Finger Windows, CPT = Continuous Performance Test, SE = standard error, RT = reaction time, ISI = inter-stimulus interval, WCST = Wisconsin Card Sorting Test, TOL = Tower of London.

Standard scores were used for all WCST and TOL measures, and T-scores were used for CPT measures (excl. overall index).

Three major haplotype blocks in *SLC6A2* are depicted above: Block 1 (rs1362621, rs2397771, rs168924, rs2242446, rs3785143, rs192303, rs41154); Block 2 (rs36017, rs10521329, rs3785155, rs5564, rs11568324, rs2279805, rs8047672, rs5569, rs998424, rs36009); and Block 3 (rs1800887, rs2242447, rs15534).

Significance (p) value ranges are depicted as follows: For OVER-TRANSMISSION of alleles = **+4** (≤0.00001), **+3** (0.0001–0.0009), **+2** (0.001–0.009), and **+1** (0.01–0.049).

For UNDER-TRANSMISSION of alleles = −**4** (≤0.00001), −**3** (0.0001–0.0009), −**2** (0.001–0.009) and −**1** (0.01–0.049).

**Table 10:** Association between *SLC6A2* SNPs and treatment response in the sample where mothers did not smoke during pregnancy.

			BLOCK 1														BLOCK 2									BLOCK 3			
TAG SNPs/HAPLOTYPES	rs28386840	rs4783899	rs1362621	rs2397771	rs168924	rs2242446	rs3785143	rs192303	rs41154	rs187715	rs36024	rs187714	rs36023	rs36021	rs3785152	rs1814269	rs36017	rs10521329	rs3785155	rs5564	rs2279805	rs8047672	rs5569	rs998424	rs36009	rs1800887	rs2242447	rs15534	rs7188230
ALLELES	A	T	A	G	C	T	C	C	G	A	C	C	C	T	C	A	G	A	A	T	C	A	G	C	G	T	T	T	A
**TREATMENT RESPONSE**																													
CGI - improvement (PBO-active)															**+3**	**+1**													
Conners’ P RI (PBO-active)							−**1**																						
Conners’ T RI (PBO-active)															**+1**				**+1**				**+1**	**+1**					
Conners’ P EL (PBO-active)																													
Conners’ T EL (PBO-active)															**+1**				**+1**	**+1**					**+1**				
Conners’ P (PBO-active)																													
Conners’ T (PBO-active)															**+1**				**+1**				**+1**	**+1**					
RASS total difference score (PBO time2-Active time2)															**+2**	**+2**													
RASS fidgeting difference score													**+2**																
RASS vocalization difference score															**+1**												**+1**		
RASS task disengagement difference score															**+3**	**+1**													

CGI = Clinical Global Impression, PBO = placebo, Conners’ P = Conners’ Parents, Conners’ T = Conners’ Teachers, RI = Restless-Impulsive, EL = Emotional Lability, RASS = Restricted Academic Situation Scale.

Three major haplotype blocks in *SLC6A2* are depicted above: Block 1 (rs1362621, rs2397771, rs168924, rs2242446, rs3785143, rs192303, rs41154); Block 2 (rs36017, rs10521329, rs3785155, rs5564, rs11568324, rs2279805, rs8047672, rs5569, rs998424, rs36009); and Block 3 (rs1800887, rs2242447, rs15534).

Significance (p) value ranges are depicted as follows: For OVER-TRANSMISSION of alleles = **+4** (≤0.00001), **+3** (0.0001–0.0009), **+2** (0.001–0.009), and **+1** (0.01–0.049).

For UNDER-TRANSMISSION of alleles = −**4** (≤0.00001), −**3** (0.0001–0.0009), −**2** (0.001–0.009) and −**1** (0.01–0.049).

### Statistical Analyses

Family-based tests of association (which examine the transmission disequilibrium of a specific allele/haplotype from parent to affected offspring) were conducted using the FBAT statistical package (version 2.0.3). [72] All analyses were performed under the assumption of an additive model, with a null hypothesis of no linkage and no association. Tests were first conducted with the total sample, and then by maternal smoking during pregnancy stratification (MSDP). Of the total number of nuclear families in the study (n = 377), we had information related to MSDP for 366 families, where 206 were coded as ‘non-smoking’ and 160 as ‘smoking’.

## Results

As noted in Tables 1, 2, and 3, marginal association was observed with several behavioral and cognitive dimensions of ADHD in the total sample. However, the most significant result was noted when FBAT analysis was conducted in the stratified group where mothers smoked during pregnancy (Table 4). Whereas a marginal association was observed with rs36021 in the total sample (*Z* = 2.54, *P* = 0.01), a highly significant association was observed on every measure tested, as well as treatment response in the stratified sample. The *T* allele of this SNP appears to be the risk allele for ADHD, showing an association with the categorical DSM-IV diagnosis (*Z* = 3.74, *P* = 0.0002). In the quantitative FBAT analysis, the *T* allele was over-transmitted to the higher number of inattention (*Z* = 3.91, *P* = 0.00009), hyperactivity (*Z* = 3.33, *P* = 0.0009), and impulsivity (*Z* = 2.93, *P* = 0.003) items on the DISC-IV, higher CBCL total scores (*Z* = 3.95, *P* = 0.00008) (as well as each of the dimensional scores), higher restless-impulsive subscale scores of Conners’-T (*Z* = 2.72, *P* = 0.006) and Conners’-P (*Z* = 2.75, *P* = 0.006). Taken together, this suggests that the *T* allele is associated with more severe psychopathology, as assessed in the home, school, and clinic.

In terms of cognitive function, the risk allele was associated with worse performance on the SOPT (*Z* = 3.69, *P* = 0.0002), CPT and WCST (Table 5). Since the SOPT score is not a standardized score, higher scores imply worse performance, i.e. poor spatial working memory, planning, and response inhibition. A highly significant association was observed with the CPT overall index (a weighted sum of all measures within the CPT) (*Z* = 3.49, *P* = 0.0005). The risk allele was over-transmitted to the higher scores, with higher *T*-scores implying worse performance. In particular, an association was noted with several dimensions evaluated in this test – hit reaction time (RT) standard error (SE) (*Z* = 3.5, *P* = 0.0005) and variability of SE (*Z* = 3.0, *P* = 0.003). High *T*-scores on these measures indicate highly variable reactions to the “target” and “non-target”, often related to inattentiveness. [73] Highly significant association was also observed with hit RT block change (*Z* = 3.74, *P* = 0.0002) and hit SE block change (*Z* = 2.86, *P* = 0.004). Here, the higher *T*-scores indicate a slowing in reaction time, as well as a loss of consistency, which together suggest a loss of vigilance, as the test progresses. The risk allele was also associated with poor performance on the WCST, which measures cognitive flexibility and set-shifting. The *T* allele showed an under-transmission (negative *Z* score) to the higher scores, specifically with non-perseverative errors (the higher standard scores imply a better performance on the test) (*Z* = -3.44, *P* = 0.0006). No association was observed with perseverative errors or responses. On the WCST, perseverative errors occur due to an inability to shift set, despite negative feedback. [56] Non-perseverative errors are incorrect categorizations not related to perseveration, and usually arise from distractibility as well as deficits in updating and monitoring working memory. Therefore, it appears that in the group where mothers smoked during pregnancy, children with the *T* allele at rs36021 exhibit EF deficits, specifically sustained attention (characterized by distractibility during the task and loss of vigilance as the test progresses), spatial working memory, planning, and response inhibition.

The *T* allele was also associated with response to MPH treatment (Table 6). The risk allele was associated with greater improvement as indexed by a higher change score (score after PBO – score after MPH) on the CGI (*Z* = 3.275, *P* = 0.001), Conners’-P (*Z* = 2.62, *P* = 0.009), as well as evaluation in the simulated academic environment, (*Z* = 3.58, *P* = 0.0003). Based on the factor structure of the RASS, [68] change scores were examined for fidgeting, vocalizing and task disengagement. Association was observed with the task disengagement factor (*Z* = 3.44, *P* = 0.0006), but not with the other factors.

FBAT analysis in the group where mothers smoked during pregnancy also showed significant association between other SNPs towards the 5′end of *SLC6A2* and one or more behavioral/cognitive measures. These included: rs41154, rs187714, and to a lesser extent, rs4783899, rs2397771, and rs192303. Based on calculation of D’ and r^2^ in Haploview, it was noted that these markers are in strong LD with rs36021 (Table 7), explaining the parallel association observed on several of the measures. Conversely, markers that are not in strong LD with rs36021 (such as rs36023 and rs36024) do not show an association with ADHD or any of the relevant dimensions in this sub-group.

In the sample where mothers did not smoke during pregnancy, marginal association with rs3785152 was observed on several behavioral and cognitive dimensions (Tables 8, 9, 10). In contrast, this SNP showed a highly significant association with treatment response. As with rs36021, the *C* allele was associated with significant improvement on behavioral evaluations; CGI_ [PBO – MPH]_ (*Z* = 3.5, *P* = 0.0005), RASS task-disengagement_ (PBO – MPH)_ (*Z* = 3.58, *P* = 0.0003). No association was observed with rs36021 in this group. It is interesting that two adjacent SNPs (rs36021 and rs3785152) show highly divergent association in the two groups. In fact, LD between these two SNPs is low (Table 7). Therefore, it is likely that a recombination event at or close to these two SNPs resulted in at least two distinct variants of *SLC6A2*. Association was also observed with rs1814269, rs5569, rs998424, and rs36009 in this group, though the significance was marginal.

## Discussion

Conducting stratified analyses based on MSDP provides great insight into the complex association between *SLC6A2* and ADHD. Although pharmacological, imaging, and neuropsychological studies have extensively implicated the norepinephrine transporter in ADHD, genetic studies have shown a minimal association. Although associations have been reported, non-replication between studies has resulted in a lack of overall significance when a meta-analysis was conducted. [74] Results presented here, and in an earlier report, [75] support the view that the lack of replication between studies may be explained, at least in part, by the inherent clinical and etiological complexity of the disorders.

The association between MSDP and ADHD is one of the most investigated in the field of environmental psychiatric epidemiology. Although consistently replicated [76], [77], [78] and high in magnitude, there is now relative consensus that this association has little causal significance [79], [80] and may instead be driven by other variables that are shared by the behavior of smoking during pregnancy in mothers and ADHD in their children. While environmental factors may play a role in this association, it is believed that genetic factors shared by mother and child play an important role in smoking during pregnancy in the former and ADHD in the latter. In this study, MSDP was used to index a subtype of ADHD with putatively more homogeneous genetic determinants shared within families of children with ADHD where mothers smoked during pregnancy. Consistent with this hypothesis, we have reported [81] that children in this subgroup present a more severe clinical picture with greater behavioral problems and lower cognitive function, when compared to children whose mothers did not smoke during pregnancy, and that this difference in clinical phenotype is significant even when important environmental factors are controlled for. The results of the current study emphasize the genetic differences in these two subtypes. Polymorphisms (rs36021 and linked SNPs) are important genetic determinants of behavior, cognition, and treatment response in ADHD children whose mothers smoked during pregnancy, and who may represent a more homogeneous group of ADHD patients, as previously reported. [81] In the subtype where mothers did not smoke during pregnancy, an association with a different region of the gene (towards the 3′ end of *SLC6A2)* is observed.

Given that the association between ADHD and rs36021 (and linked SNPs) is highly significant only in those children whose mothers smoked during pregnancy may suggest a true interaction between exposure to maternal smoking and carrying the risk allele(s) in the SLC6A2 gene. Indeed, the adverse consequences of *in utero* exposure to the toxic effects of nicotine are well documented, from animal and human studies. [82] MSDP is associated with pre- and peri-natal complications, deficits in cognitive development as well as long-term behavioral problems. Alternatively, but not exclusively, the etiology of smoking behavior and ADHD may involve closely related, but distinct pathways. Indeed, it is possible that the complex genetic background underlying smoking behaviors in mothers (which is transmitted in part to their children), interacts with risk alleles in *SLC6A2* to increase the risk for ADHD in children. Under the latter scenario, MSDP may be considered as a phenotypic index used to select a subgroup of children with relatively more homogeneous genetic etiology.

Irrespective of the precise links between these pathways, this study strongly suggests that genetic variation in the *SLC6A2* is an important factor in a more severe subtype of ADHD. If replicated in independent studies, this may represent an important step towards *personalized medicine* in treating children with ADHD. [83].

Results of the present study are perfectly congruent with reports by Song *et al* [35] and Yang *et al*, [40] but only in the group where mothers did not smoke during pregnancy. In this group, a significant over-transmission of the *G* allele to the higher difference scores was observed in the quantitative FBAT analysis on the Conners’-T (Table 10). Most of this effect appears to arise from the restless-impulsive factor scores, observed only in the group of non-smoking mothers. It is noted that when treatment response was assessed using the CGI-Improvement scale, two previous studies, [38], [42] as well as the current study, did not find an association with 1287(G/A) (rs5569) (Table 10).

Several other previously-reported associations were replicated in the present study. Three studies had reported an association with rs3785143 and rs11568324. [23], [26], [29] These markers are in complete LD with rs36021 (*D*’ = 1; albeit with a low correlation coefficient, *r* ^2^; Table 7), indicating that the 3 SNPs are in one haplotype block not separated by a recombination event. In the total sample, rs3785143 showed marginal association with ADHD, but a significant association with all CBCL dimensional scores (Table 1). No association was observed when stratified analyses were carried out. Similarly, no association was observed with rs11568324 despite the fact that it is in complete LD with rs36021. This is most likely a result of the low heterozygosity of these markers, which make them non-informative in the FBAT analysis (as indicated by the number of informative families in Table 1). Two other previously-implicated SNPs, rs998424 and rs36017, showed marginal association with dimensions of ADHD in the sample where mothers did not smoke during pregnancy and the total sample, respectively.

Kim and colleagues [31], [32], [33] reported an association between ADHD and a functional promoter SNP rs28386840 [-3081(A/T)] in several independent case-control studies. This association was not replicated in the current study, neither in the total sample, nor in the samples stratified by MSDP (Tables 1, 4, 8). The lack of association with ADHD was also reported in two other family-based studies. [24], [28] A study examining the association between this polymorphism and treatment response reported an association with CGI-improvement scores [38], where *T*-allele carriers showed a better response to MPH treatment. In the current study, only a marginal association was observed with difference scores on the restless-impulsive subscale of the Conners’-T in the group where mothers smoked during pregnancy (Table 6).

In a previous report, [47] we investigated the association between ADHD and the panel of 30 SNPs examined in the present study, and noted that a complex pattern of association emerged between *SLC6A2* SNPs/haplotypes, ADHD subtypes and gender. Gender and subtype are considered two dimensions that might help in reducing genetic heterogeneity in the ADHD syndrome. Although these results helped explain some of the discrepancies noted among previous studies, stratification according to these dimensions did not yield as strong an association with *SLC6A2* as the stratification based on MSDP, which may suggest that the latter is more pertinent for future efforts to map genes implicated in ADHD.

SNPs that showed the most significant association in this study (rs36021 and rs3785152, in particular) are within introns, opening up two possibilities. The first possibility is that these intronic variants are involved in gene regulation. The second is that these polymorphisms are not the causal mutation, but are in LD with a functional variant. Fine-mapping of the region is required to identify the causal mutation(s) followed by molecular analysis to determine if the mutation affects transcriptional regulation of the gene or structure and function of the protein.

While we conducted a large number of comparisons and some correction for multiple testing is warranted, it is important to note that when we correct for multiple testing in relation to our primary hypothesis, that is association between *SLC6A2* and ADHD in children stratified according to MSDP, the primary result of association (*Z* = 3.74, *P* = 0.0002) with rs36021 remains significant even if we apply the overly stringent Bonferroni correction (30 SNPs times two exposure strata, p = 0.002). In addition, the widespread exploratory associations that are observed with behaviors relevant to ADHD measured by different observers (parents, teachers, and research staff) and in different settings (school, home, clinic) with rs36021 suggest that these associations are unlikely to be chance findings. We believe that this considerable consistency of results strengthens the overall credibility of the primary results and help to understand how genetic vulnerability to ADHD is mediated through the traits and endophenotypes underlying this disorder.

To our knowledge, this is the largest study (among family-based and case-control studies) testing the association between ADHD and *SLC6A2*, with such detailed genotype and phenotype characterization. While collaboration between multiple research groups in large consortia is vital for genetic studies of ADHD, it has been shown that a significant amount of heterogeneity can be introduced in multicenter collaborative studies because of divergent clinical and evaluation practices. [84] This underscores the value of the current study where a relatively large sample has been collected at a single center using a highly standardized approach. It is also the largest study worldwide to use a double-blind, placebo-controlled design for evaluation of treatment response, combining extensive evaluation of executive function and behavioral domains, with genetic and environmental data. Nonetheless, these results must be considered exploratory and independent replication is awaited.

If confirmed in independent studies, these results will help to disentangle the complex etiological pathways of ADHD. In the long term, this would very likely lead to development of therapeutics targeting specific biochemical pathways in specific sub-groups of children with ADHD.
